# Mathematical analysis of *Mycoplasma Genitalium* transmission dynamics: A compartmental model with nonlinear interactions and treatment effects

**DOI:** 10.1371/journal.pgph.0006834

**Published:** 2026-07-27

**Authors:** Bolarinwa Bolaji, Benjamin Idoko Omede, Benedict Celestine Agbata, Godwin Onuche Acheneje, Homan Emadifar, Aseel Smerat

**Affiliations:** 1 Mathematical Sciences Department, Prince AbubakarAudu (Formerly Kogi State) University, Anyigba, Nigeria; 2 Laboratory of Mathematical Epidemiology and Applied Sciences (LOMEAS), Mathematical Sciences Department, Prince Abubakar Audu (Formerly Kogi State) University, Anyigba, Nigeria; 3 Mathematics Department and Statistics, Confluence University of Science and Technology, Osara, Nigeria; 4 Department of Mathematics, Saveetha School of Engineering, Saveetha Institute of Medical and Technical Sciences, Saveetha University, Chennai, Tamil Nadu, India; 5 Department of Basic Sciences, Technical and Vocational University (TVU), Tehran, Iran; 6 Hourani Center for Applied Scientific Research, Al-Ahliyya Amman University, Amman, Jordan; 7 Centre for Research Impact and Outcome, Chitkara University, Punjab, India; PLOS: Public Library of Science, UNITED STATES OF AMERICA

## Abstract

Due to the increasing resistance to drug treatment among individuals infected with *Mycoplasma Genitalium*, it is essential to incorporate this factor into its mathematical modelling with a view to gaining insights into how to effectively combat the spread of the disease. To this extent, consequently, in this study, we developed a deterministic mathematical model that considers two strains of the disease: the drug-sensitive and drug-resistant strains. The analysis of the treatment model revealed that its disease-free equilibrium is globally asymptotically stable when a certain epidemiological threshold $\left(R_{\text{0}}^{t}\right)$is less than one, indicating disease control. However, when this threshold exceeds one, $R_{\text{0}}^{t}>1$, the model exhibits two co-existing endemic equilibria, and competitive exclusion occurs if the reproduction number of the drug-resistant strain is higher than that of the drug-sensitive strain. In contrast, the model without treatment shows infinitely many endemic equilibria when the epidemiological threshold exceeds one. Furthermore, it was discovered that widespread use of antibiotics to treat the wild strain of the disease can eliminate it from the community if the reduction in infectiousness does not exceed a certain value. Sensitivity analysis of the model helped identified key parameters influencing the disease transmission dynamics, which were supported by numerical simulations. Based on our findings, we recommend effective strategies to policymakers in the healthcare sector for combating the spread of *M. Genitalium* and mitigating its burden.

## 1 Introduction

Mycoplasmas are the smallest self-replicating microorganisms and *M. Genitalium* is recognized as the smallest prokaryote capable of self-replication. It belongs to the class Mollicutes in the family Mycoplasmataceae. In 1980, *M. Genitalium* was first isolated from urethral swabs of two symptomatic men with non-gonococcal urethritis (NGU) [1]. Unlike other sexually transmitted infections (STIs) such as Chlamydia trachomatis, there have been limited studies on the prevalence of *M. Genitalium*, primarily due to its challenging nature to culture [2,3]. Infections caused by *M. Genitalium* can be chronic, symptomatic, or asymptomatic. The primary mode of transmission is through direct genital mucosal contact during unprotected sexual intercourse. The incubation period for *M. Genitalium* ranges from two to thirty-five days [4]. Conventional culture techniques struggle to isolate the bacterium from clinical specimens, hence the diagnosis relies on sensitive Nucleic Acid Amplification Tests (NAATs) [5]. Although transmission through penile-anal intercourse is possible (popularized by the belief that it reduces the risk of pregnancy but increases STI and HIV infection), studies indicate that oral-genital intercourse is less likely to contribute significantly to transmission, as the carriage of *M. Genitalium* in the oropharynx is rare [5]. While mother-to-child transmission at birth has not been extensively studied, *M. Genitalium* has been detected in the respiratory tracts of newborns [4].

*M. Genitalium* is associated with various pathological conditions and is particularly linked to sexually transmitted infections (STIs). The clinical manifestations of *M. Genitalium* infection differ between men and women. Studies indicate that 40–75% of women attending STI clinics are asymptomatic, while 70% of men experience symptoms [1,5]. In women, symptoms can include increased or altered vaginal discharge (more than 50% of normal), inter-menstrual or postcoital bleeding, and symptoms related to cervical and urethral infection [6–8]. Rectal and pharyngeal infections are often asymptomatic. Symptoms such as dysuria, urgency, cervicitis, pain during intercourse, bleeding after intercourse, stinging or burning during urination, and lower abdominal pain may indicate the presence of Pelvic Inflammatory Disease (PID) [1,4,5]. *M. Genitalium* has been associated with urogenital consequences such as male and female urethritis, balanoposthitis, prostatitis, cervicitis, PID, and infertility in both men and women [9]. The most common manifestation in men is acute or chronic urethritis, which can cause dysuria, balanoposthitis, prostatitis, urethral discharge, and proctitis. Transmission of *M. Genitalium* during pregnancy may slightly increase the risk of spontaneous abortion and preterm birth. Untreated *M. Genitalium* infections can lead to complications [9–12].

Treatment of *M. Genitalium* poses challenges due to macrolide resistance in some patients. Oral antibiotics are used for treatment, with Azithromycin and Moxifloxacin being the preferred choices. However, resistance to Moxifloxacin has also been observed [10]. Resistance to fluoroquinolones is associated with mutations in the gyrA and parCgenes [11]. Some strains of *M. Genitalium* may be resistant to standard treatment, requiring additional antibiotics [10]. Tetracycline was initially used for its treatment but showed poor efficacy. Azithromycin, given as a single 1g dose, has demonstrated unsatisfactory efficacy in recent years, possibly due to increased macrolide resistance. Macrolide-susceptible infections have a cure rate of 85–95% with Azithromycin. Macrolide-resistant strains are currently managed with Moxifloxacin, although resistance to this fluoroquinolone has also been observed, especially in the Asia-Pacific region. The emergence of new mutants is attributed to factors such as incorrect antibiotic regimens, certain biological factors, and primary infection with drug-resistant strains of the disease [2,10,5,12].

Mathematical models play a crucial role in understanding the transmission dynamics of contagious diseases and devising effective control strategies, such works as those in [13–34]. While numerous studies have focused on the clinical aspects of *M. Genitalium*, such as those in [7,35–37], there is a lack of literature on mathematical modeling of its transmission dynamics, possibly due to its re-emerging nature. Therefore, the aim of this study is to bridge this knowledge gap by formulating and rigorously analyzing a deterministic model that incorporates both the drug-sensitive and drug-resistant strains of the disease, considering the impact of antibiotic treatment. This model aims to provide a foundation for understanding the transmission dynamics and exploring strategies for controlling the spread of *M. Genitalium*.

The article is organized as follows: In Sect 2, we presented the model formulation, including the assumptions and flow diagram. In Sect 3 examined the dynamical features of the model, demonstrating that the state variables are non-negative and analyzing the local and global asymptotic stability of the steady state. In Sect 4, we conducted sensitivity analysis to identify the most influential parameters on the transmission dynamics of the disease. In Sect 5 we presented numerical simulations of the model so as to validate the theoretical results obtained earlier. Finally, in Sect 6, we summarized the findings, provide recommendations to policymakers in the healthcare sector for combating the spread of the disease, and discuss potential ways to mitigate the disease burden.

## 2 Model formulation

In this study, the total population of the community at a given time is divided into distinct compartments, each representing different groups of individuals. These compartments include:

Susceptible individuals, S(t), are those who have not yet encountered the pathogen and can still contract it. Exposed individuals with the wild strain, $E_{W}\left(t\right)$, have encountered the wild strain but are not yet infectious. Exposed individuals with the resistant strain, $E_{R}\left(t\right)$, have encountered the resistant strain but are likewise not yet infectious. Asymptomatic infected individuals with the wild strain, $I_{AW}\left(t\right)$, carry the wild strain without showing symptoms. Symptomatic infected individuals with the wild strain, $I_{SW}\left(t\right)$, carry the wild strain and do show symptoms. Asymptomatic infected individuals with the resistant strain, $I_{AR}\left(t\right)$, carry the resistant strain without symptoms. Symptomatic infected individuals with the resistant strain, $I_{SR}\left(t\right)$, carry the resistant strain and present symptoms. Treated individuals, T(t), have received therapy. Recovered individuals, R(t), have cleared the infection and gained immunity. Consequently, the total population of the inhabitants of the geographical location under reference at any given time is the sum of individuals in the afore-mentioned compartments, given as:


$$\begin{array}{c} N\left(t\right)=S\left(t\right)+E_{W}\left(t\right)+E_{R}\left(t\right)+I_{AW}\left(t\right)+I_{SW}\left(t\right)+I_{AR}\left(t\right)+I_{SR}\left(t\right)+T\left(t\right)+R\left(t\right)\text{.} \end{array}$$

The susceptible population class (S(t)), are populated by the recruitment of individuals, the newborns and immigrants at a rate π and $\lambda_{W}$ is the rate at which susceptible individuals acquire infection when they come in contact with person infected with the wild strain of the disease and $\lambda_{R}$, is the rate of primary infection with resistant strain of the disease. The exposed individuals in classes $\left(E_{W}\left(t\right)\right)$ and $\left(E_{R}\left(t\right)\right)$ progressed to infected class with the wild strain of the disease at the rate $\sigma_{W}$and $\sigma_{R}$ for those that moved to infected class with wild and resistant strainrespectively. The fraction, $f_{\text{1}}$, $f_{\text{2}}$ where $0<f_{\text{1}}<1$ and $0<f_{\text{2}}<1$ are for individuals that progressed from exposed with wild (resistant) strain to asymptotically infected with the wild (resistant) strain at the rate $\left(1-f_{\text{1}}\right)\sigma_{W}$, $f_{\text{1}}\sigma_{W}$and ($f\sigma_{R}$, $\left(1-f\right)\sigma_{R}$) respectively. The rate $\gamma_{W}$($\gamma_{R}$) is of progression to symptomatic infected class $I_{SW}$($I_{SR}$) with the wild (resistant) strain from asymptomatic infected class$I_{AW}$ ($I_{AR}$) respectively. The rate $\tau_{W}$ is of progression to treatment class from symptomatic class $I_{SW}$, while mortality rate is μwhich is the same in all compartments. Those who progressed to the class of symptomatic infected with wild (resistance) strain do so at the rate $\gamma_{W}$($\gamma_{R}$) respectively. The fraction q, where 0<q<1 is that of individuals who resisted treatment in the class for those that are treated for the disease at the rate qϕ (a proportion of this goes to $\left(I_{AR}\left(t\right)\right)$ class and the remaining goes to $\left(I_{SR}\left(t\right)\right)$ class), where ε denotes the rate at which individuals under treatment clear the infection and move to the recovered class.

**Fig 1:**
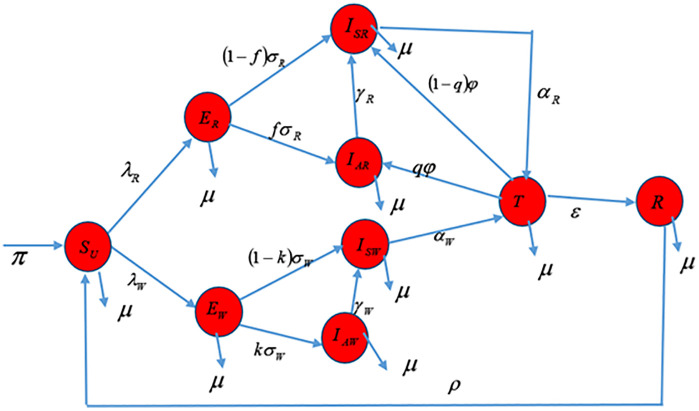
Compartmental diagram for *Mycoplasma Genitalium* model (1).

Based on the above formulations and assumptions, we obtained the following system of non-linear differential equations governing the transmission dynamics of *M. Genitalium* with a schematic diagram depicted by Fig 1; with state variables and parameters used in the model building described in Table 1.

**Table 1:** Interpretation of symbols used as variables and parameters for model (1).

Variables	Interpretation
S	Individuals that are susceptible to the disease
$E_{W}$	Individuals that are exposed to the Wild strain of the disease
$E_{R}$	Individuals that are exposed to the Resistance strain of the disease
$I_{AW}$	Individuals that are infected asymptomatic with Wild strain
$I_{SW}$	Infected symptomatic individuals with Wild strain
$I_{AR}$	Infected asymptomatic individuals with Resistance strain
$I_{SR}$	Infected symptomatic individuals with Resistance strain
T	Treated individuals
R	Recovered individuals
**Parameters**	**Interpretation**
π	Recruitment rate
$\beta_{W},\beta_{R}$	Transmission rate for Wild and Resistance strain respectively
$\sigma_{W},\sigma_{R}$	Progression rate from exposed to infected for wild and resistance respectively
$\gamma_{W},\gamma_{R}$	Progression rate from infected Asymptomatic to symptomatic wild (resistance) strain of the disease
κ	A fraction that progressed to infected Asymptomatic Wild strain
f	A fraction that progressed to infected Asymptomatic Resistance strain
$\alpha_{W},\alpha_{R}$	Progression from Wild and Resistance infected to Treated compartment respectively
ε	Rate of recovery of treated individuals
φ	Drug-resistant rate after treatment.
q	Fraction of individuals that resist treatment
μ	Natural death rate
$\lambda_{W},\lambda_{R}$	Force of infection for Wild and Resistance strain respectively
ρ	Treatment-induced immunity loss rate


$$\begin{array}{c} \frac{dS}{dt}=\pi +\rho R-\left(\lambda_{W}+\lambda_{R}\right)S-\mu S\text{,} \end{array}$$

$$\begin{array}{c} \frac{dE_{W}}{dt}=\lambda_{W}S-\left(\sigma_{W}+\mu \right)E_{W}\text{,} \end{array}$$

$$\begin{array}{c} \frac{dE_{R}}{dt}=\lambda_{R}S-\left(\sigma_{R}+\mu \right)E_{R}\text{,} \end{array}$$

$$\begin{array}{c} \frac{dI_{AW}}{dt}=k\sigma_{W}E_{W}-\left(\gamma_{W}+\mu \right)I_{AW}\text{,} \end{array}$$ (1)

$$\begin{array}{c} \frac{dI_{SW}}{dt}=\left(1-k\right)\sigma_{W}E_{W}+\gamma_{W}I_{AW}-\left(\alpha_{W}+\mu \right)I_{SW}\text{,} \end{array}$$

$$\begin{array}{c} \frac{dI_{AR}}{dt}=f\sigma_{R}E_{R}-\left(\gamma_{R}+\mu \right)I_{AR}+q\varphi T\text{,} \end{array}$$

$$\begin{array}{c} \frac{dI_{SR}}{dt}=\left(1-f\right)\sigma_{R}E_{R}+\gamma_{R}I_{AW}-\left(\alpha_{R}+\mu \right)I_{SR}+\left(1-q\right)\varphi T\text{,} \end{array}$$

$$\begin{array}{c} \frac{dT}{dt}=\alpha_{W}I_{SW}+\alpha_{R}I_{SR}-\left(\varepsilon +\varphi +\mu \right)T, \end{array}$$

$$\begin{array}{c} \frac{dR}{dt}=\varepsilon T-(\mu +\rho )R, \end{array}$$

Where $\lambda_{W}=\frac{\beta_{W}\left(I_{AW}+I_{SW}\right)}{N}$ and $\lambda_{R}=\frac{\beta_{R}\left(I_{AR}+I_{SR}\right)}{N}$

In the formulation of the model, some assumptions were made viz:

(i) There is uniform rate of natural death for all individuals in all the compartments of the model.
(ii) There is resistant to treatment using antibiotics, thus two strains of the disease exist, the wild and the drug-resistant strain as obtained in the literature [11].
(iii) Treatment of infected individuals only confers temporary immunity against the disease as reinfection can take place after treatment [20,21,26].
(iv) There is no disease-induced death since the disease cannot cause death [2,10,38,39].
(v) There is no natural recovery for infected humans.

Fig 1 serves a crucial role in the mathematical modelling *M. Genitalium* transmission dynamics by visually representing the structure and dynamics of disease transmission [29,33,35]. It simplifies complex systems into intuitive components such as compartments and the interactions between them, often depicted as arrows indicating transitions or rates of change [40]. This visual framework helps clarify assumptions, identify key parameters, and communicate the flow of infections within a population, such as the effects of contact rates, recovery, and immunity.

Table 1 provides a detailed interpretation of the symbols used as variables and parameters in Model [34]. Each symbol is defined with its description, applicable units, and any relevant notes that clarify its role within the model. The table is structured to enhance understanding of how each component contributes to the model’s formulation and application [40].

Next is to rigorously analyze model (1) so as to have understanding of its dynamical features. This we do in section 3as follows.

### 2.1 Dynamical features of the model

In order to ensure the epidemiological relevance of the model (1), it is crucial to establish that all its state variables remain non-negative at all time (*t*). Since the model represents the dynamics of a human population, it is necessary to demonstrate that the solutions of model (1) with positive initial data will remain positive throughout time (*t*). This ensures that the population sizes in each compartment, will always remain non-negative and reflect realistic scenarios. By establishing the non-negativity of the solutions, we can ensure the meaningfulness and validity of the epidemiological interpretations derived from the model.

### 2.2 Positivity of solution

**Theorem 2.1**

Suppose that initial data be $\left\{\left(S\left(0\right),E_{W}\left(0\right),E_{R}\left(0\right),I_{AW}\left(0\right),I_{SW}\left(0\right),I_{AR}\left(0\right),I_{SR}\left(0\right),T\left(0\right),R\left(0\right)\right)\geq 0\right\}\in R_{+}^{\text{9}}$.

Then solution set $\left\{\left(S\left(t\right),E_{W}\left(t\right),E_{R}\left(t\right),I_{AW}\left(t\right),I_{SW}\left(t\right),I_{AR}\left(t\right),I_{SR}\left(t\right),T\left(t\right),R\left(t\right)\right)\right\}$ of the model (1) having positive initial data, will remain so for all time t>0.

**Proof:**

For model (1), we consider its first equation:


$$\begin{array}{c} \frac{dS}{dt}=\pi +\rho R-\left(\lambda_{W}+\lambda_{R}\right)S-\mu S, \end{array}$$

$$\begin{array}{c} \frac{dS}{dt}=\pi +\rho R-\left[\lambda_{W}+\lambda_{R}+\mu \right]S\geq -\left[\lambda_{W}+\lambda_{R}+\mu \right]S\text{.} \end{array}$$

By integrating both sides of this, we have:


$$\begin{array}{c} \int \frac{dS}{S}\geq \int -\left[\lambda_{W}+\lambda_{R}+\mu \right]dt\text{,} \end{array}$$

$$\begin{array}{c} ln\;S\geq -\left[\lambda_{W}+\lambda_{R}+\mu \right]t+c_{\text{1}}\text{.} \end{array}$$

The exponential of both sides when taken gives:


$$\begin{array}{c} S\left(t\right)\geq e^{-\left[\lambda_{W}+\lambda_{R}+\mu \right]t+c_{\text{1}}}\text{,} \end{array}$$

$$\begin{array}{c} S\left(t\right)\geq c_{\text{1}}e^{-\left[\lambda_{W}+\lambda_{R}+\mu \right]t}\text{.} \end{array}$$

adopting the initial condition t=0, $S\left(0\right)\geq c_{\text{1}}$, gives:


$$\begin{array}{c} S\left(t\right)\geq S\left(0\right)e^{-\left[\lambda_{W}+\lambda_{R}+\mu \right]t}>0,\text{\textasciitilde{}Since\textasciitilde{}}\left[\lambda_{W}+\lambda_{R}+\mu \right]>0. \end{array}$$

In the same way, we show that other state variables:


$$\begin{array}{c} S\left(t\right)>0,E_{W}\left(t\right)>0,E_{R}\left(t\right)>0,I_{AW}\left(t\right)>0,I_{SW}\left(t\right)>0,I_{AR}\left(t\right)>0,I_{SR}\left(t\right)>0,T\left(t\right)>0\text{\textasciitilde{}and\textasciitilde{}}R\left(t\right)>0. \end{array}$$

Arising from the above, all solutions of the model (1) will remain non-negative at all times for all non-negative initial conditions.

**Lemma 1:** At all times, there exist feasible solutions of the equations in model (1) if they are contained in the following invariant region D:


$$\begin{array}{c} D=\left\{\left(S,E_{W},E_{R},I_{AW},I_{SW},I_{AW},I_{AR},T,R\right)\in R_{+}^{\text{9}}:N\leq \pi \;/\;\mu \right\} \end{array}$$

**Proof.** On adding all the equations contained in the model (1) leads to:


$$\begin{array}{c} \frac{dN}{dt}=\pi -\mu N \end{array}$$ (2)

Hence, given that N>π/μ, then dN/dt<0. Consequently, arising from the fact that dN/dt is bounded by π−μN, we can use a standard comparison theorem [41] to generate


$$\begin{array}{c} N\leq N\left(0\right)e^{-\mu t}+\pi \;/\;\mu \left(1-e^{-\mu t}\right) \end{array}$$

Given that N(0)≤π/μ, then N(t)≤π/μ. Consequently, the set D, defined by the model (1), is proven to be positively-invariant under the flow of the system. This means that no solution path deviates from D through any of its boundaries. Consequently, it is appropriate to analyze the dynamics of the model (1) within the region D. By focusing on this region, we ensure that the model is both epidemiologically and mathematically well-posed, as established by previous research [42–44]. Considering the dynamics within D allows us to accurately study the behavior and outcomes of the model, ensuring its validity and relevance in both epidemiological and mathematical contexts.

### 2.3 Treatment-free model

For convenience, it is imperative to first study a version of model (1) when treatment is made insignificant, herein typifies has treatment-free model which is obtained by setting $T=R=\alpha_{W}=\alpha_{R}=\phi =\varepsilon =0$ before we proceed to analyze the treatment model (1); given by:


$$\begin{array}{c} \frac{dS}{dt}=\pi +\rho S-\left(\lambda_{W}+\lambda_{R}\right)S-\mu S\text{,} \end{array}$$

$$\begin{array}{c} \frac{dE_{W}}{dt}=\lambda_{W}S-\left(\sigma_{W}+\mu \right)E_{W}\text{,} \end{array}$$

$$\begin{array}{c} \frac{dE_{R}}{dt}=\lambda_{R}S-\left(\sigma_{R}+\mu \right)E_{R}\text{,} \end{array}$$

$$\begin{array}{c} \frac{dI_{AW}}{dt}=k\sigma_{W}E_{W}-\left(\gamma_{W}+\mu \right)I_{AW}\text{,} \end{array}$$ (3)

$$\begin{array}{c} \frac{dI_{SW}}{dt}=\left(1-k\right)\sigma_{W}E_{W}+\gamma_{W}I_{AW}-\mu I_{SW}\text{,} \end{array}$$

$$\begin{array}{c} \frac{dI_{AR}}{dt}=f\sigma_{R}E_{R}-\left(\gamma_{R}+\mu \right)I_{AR}\text{,} \end{array}$$

$$\begin{array}{c} \frac{dI_{SR}}{dt}=\left(1-f\right)\sigma_{R}E_{R}+\gamma_{R}I_{AR}-\mu I_{SR}\text{,} \end{array}$$

Where $\lambda_{W}=\frac{\beta_{W}\left(I_{AW}+I_{SW}\right)}{N}$ and $\lambda_{R}=\frac{\beta_{R}\left(I_{AR}+I_{SR}\right)}{N}$.

For the treatment-free model (3), it has been demonstrated that a specific region is positively invariant and exhibits an attracting behavior. This region is defined as follows:


$$\begin{array}{c} D_{\text{1}}=\left\{\left(S,E_{W},E_{R},I_{AW},I_{SW},I_{AR},I_{SR}\right)\in R_{+}^{\text{7}}:S+E_{W},E_{R}+I_{AW}+I_{SW}+I_{AR}+I_{SR}\leq \pi \;/\;\mu \right\}, \end{array}$$

By establishing the positivity and attractiveness of a specific region, it is possible to focus solely on the dynamics of the treatment-free model (3) within this region $D_{\text{1}}$. Therefore, it is adequate to analyze and interpret the behavior of the model within this particular region [45,46]. This simplifies the study of the treatment-free model, allowing for a more focused and insightful analysis of its dynamics.

#### 2.3.1 Local stability of the disease-free equilibrium.

The disease-free equilibrium of the treatment-free model (3) is:


$$\begin{array}{c} \varepsilon_{\text{0}}=\left(S^{*},E_{W}^{*},E_{R}^{*},I_{AW}^{*},I_{SW}^{*},I_{AR}^{*},I_{SR}^{*}\right)=\left(\pi \;/\;\mu ,0,0,0,0,0,0\right) \end{array}$$ (4)

The linear stability of equilibrium point of the model can be examined by applying the next generation operator method to the system (3). Following the notation used in [41], we define the matrices *F*and*V* to represent the new infection terms and the remaining transfer terms, respectively. These matrices are given by:


$$\begin{array}{c} F=\left[\begin{array}{cccccc} 0 & 0 & \beta_{W} & \beta_{W} & 0 & 0\\ 0 & 0 & 0 & 0 & \beta_{R} & \beta_{R}\\ 0 & 0 & 0 & 0 & 0 & 0\\ 0 & 0 & 0 & 0 & 0 & 0\\ 0 & 0 & 0 & 0 & 0 & 0\\ 0 & 0 & 0 & 0 & 0 & 0 \end{array}\right]\text{\textasciitilde{}and\textasciitilde{}}V=\left[\begin{array}{cccccc} P_{\text{1}} & 0 & 0 & 0 & 0 & 0\\ 0 & P_{\text{2}} & 0 & 0 & 0 & 0\\ -k\sigma_{W} & 0 & P_{\text{3}} & 0 & 0 & 0\\ -P_{\text{4}} & 0 & -\gamma_{W} & \mu & 0 & 0\\ 0 & -f\sigma_{R} & 0 & 0 & P_{\text{5}} & 0\\ 0 & P_{\text{6}} & 0 & 0 & -\gamma_{R} & \mu \end{array}\right]\text{.} \end{array}$$

Where $P_{\text{1}}=\left(\sigma_{W}+\mu \right)$, $P_{\text{2}}=\left(\sigma_{R}+\mu \right)$, $P_{\text{3}}=\left(\gamma_{W}+\mu \right)$, $P_{\text{4}}=\left(1-k\right)\sigma_{W}$, $P_{\text{5}}=\left(\gamma_{R}+\mu \right)$ and $P_{\text{6}}=\left(1-f\right)\sigma_{R}$

Arising from here, the basic number, typifies by: $R_{\text{0}}=\rho \left(FV^{-1}\right)$, gives:


$$\begin{array}{c} R_{\text{0}}=max\left\{R_{R},R_{W}\right\}\text{,} \end{array}$$

With $R_{R}=\frac{\beta_{R}\left(P_{\text{5}}P_{\text{6}}+\mu f\sigma_{R}+\gamma_{R}f\sigma_{R}\right)}{\mu P_{\text{2}}P_{\text{5}}}$ and $R_{W}=\frac{\beta_{W}\left(P_{\text{3}}P_{\text{4}}+\mu k\sigma_{W}+\gamma_{W}k\sigma_{W}\right)}{\mu P_{\text{1}}P_{\text{3}}}$

**Lemma 2:** Whenever $R_{\text{0}}<1$ for the treatment-free model (3), its disease-free equilibrium (DFE) given by Eq (4), is locally asymptotically stable (LAS) and it will be unstable whenever $R_{\text{0}}>1$.

It is important to highlight that $R_{W}$ and $R_{R}$ represent the basic reproduction numbers for the wild and resistant strains of the disease respectively. These reproduction numbers serve as key indicators of the disease's potential for spreading within a population. The basic reproduction number $R_{\text{0}}$ specifically quantifies the expected number of secondary infections resulting from a single primary infection introduced into a completely susceptible population. It is a valuable parameter for assessing the stability of the disease-free equilibrium and estimating the peak and final size of an epidemic.

Biologically, Lemma 2.2 implies that the elimination of *M. Genitalium* from the community (when $R_{\text{0}}<1$) is possible if the initial sizes of the subpopulations of the model fall within the basin of attraction of the Disease Free Equilibrium (DFE).

To ensure that the elimination of the disease is not dependent on the initial sizes of the subpopulations, it is crucial to demonstrate that the Disease Free Equilibrium (DFE) is globally asymptotically stable (GAS) in$D_{\text{1}}$. This global stability property is established as follows:

#### 2.3.2 Global stability of Disease Free Equilibrium (DFE).

**Theorem 1:** Whenever $R_{\text{0}}\leq 1$, the disease-free equilibrium of the model (3), represented by Eq (4), is globally asymptotically stable in $D_{\text{1}}$.

**Proof:** Given the Lyapunov function:


$$\begin{array}{c} F=\left(P_{\text{3}}P_{\text{4}}+k\sigma_{W}\left(\gamma_{W}+\mu \right)\right)E_{W}+P_{\text{1}}\left(\gamma_{W}+\mu \right)I_{AW}+P_{\text{1}}P_{\text{3}}I_{SW}+\left(P_{\text{5}}P_{\text{6}}+f\sigma_{R}\left(\gamma_{R}+\mu \right)\right)E_{R} \end{array}$$

$$\begin{array}{c} +P_{\text{2}}\left(\gamma_{R}+\mu \right)I_{AR}+P_{\text{2}}P_{\text{5}}I_{SR} \end{array}$$

And Lyapunov derivative (where a dot stands for differentiation with respect to time)


$$\begin{array}{c} F=\left(P_{\text{3}}P_{\text{4}}+k\sigma_{W}\left(\gamma_{W}+\mu \right)\right)E_{W}+P_{\text{1}}\left(\gamma_{W}+\mu \right)I_{AW}+P_{\text{1}}P_{\text{3}}I_{SW}+\left(P_{\text{5}}P_{\text{6}}+f\sigma_{R}\left(\gamma_{R}+\mu \right)\right)E_{R} \end{array}$$

$$\begin{array}{c} +P_{\text{2}}\left(\gamma_{R}+\mu \right)I_{AR}+P_{\text{2}}P_{\text{5}}I_{SR} \end{array}$$

By putting the values of ${\dot{E}}_{W}$, ${\dot{E}}_{R}$, etc. into this, we obtain:


$$\begin{array}{c} F=\left(P_{\text{3}}P_{\text{4}}+k\sigma_{W}\left(\gamma_{W}+\mu \right)\right)\left(\lambda_{W}S-P_{\text{1}}E_{W}\right)+P_{\text{1}}\left(\gamma_{W}+\mu \right)\left(k\sigma_{W}E_{W}-P_{\text{3}}I_{AW}\right)+P_{\text{1}}P_{\text{3}}\left(P_{\text{4}}E_{W}+\gamma_{W}I_{AW}-\mu I_{SW}\right) \end{array}$$

$$\begin{array}{c} +\left(P_{\text{5}}P_{\text{6}}+f\sigma_{R}\left(\gamma_{R}+\mu \right)\right)\left(\lambda_{R}S-P_{\text{2}}E_{R}\right)+P_{\text{2}}\left(\gamma_{R}+\mu \right)\left(f\sigma_{R}E_{R}-P_{\text{5}}I_{AR}\right)+P_{\text{2}}P_{\text{5}}\left(P_{\text{6}}E_{R}+\gamma_{R}I_{AR}-\mu I_{SR}\right) \end{array}$$

$$\begin{array}{c} =\left(P_{\text{3}}P_{\text{4}}+k\sigma_{W}\left(\gamma_{W}+\mu \right)\right)\lambda_{W}S-\mu P_{\text{1}}P_{\text{3}}\left(I_{AW}+I_{SW}\right)+\left(P_{\text{5}}P_{\text{6}}+f\sigma_{R}\left(\gamma_{R}+\mu \right)\right)\lambda_{R}S-\mu P_{\text{2}}P_{\text{5}}\left(I_{AR}+I_{SR}\right) \end{array}$$

$$\begin{array}{c} =\left(P_{\text{3}}P_{\text{4}}+k\sigma_{W}\left(\gamma_{W}+\mu \right)\right)\lambda_{W}S-\mu P_{\text{1}}P_{\text{3}}N\lambda_{W}+\left(P_{\text{5}}P_{\text{6}}+f\sigma_{R}\left(\gamma_{R}+\mu \right)\right)\lambda_{R}S-\mu P_{\text{2}}P_{\text{5}}N\lambda_{R} \end{array}$$

$$\begin{array}{c} =\mu P_{\text{1}}P_{\text{3}}N\lambda_{W}\left(\frac{\left(P_{\text{3}}P_{\text{4}}+k\sigma_{W}\left(\gamma_{W}+\mu \right)\right)S}{\mu P_{\text{1}}P_{\text{3}}N}-1\right)+\mu P_{\text{2}}P_{\text{5}}N\lambda_{R}\left(\frac{\left(P_{\text{5}}P_{\text{6}}+f\sigma_{R}\left(\gamma_{R}+\mu \right)\right)S}{\mu P_{\text{2}}P_{\text{5}}N}-1\right) \end{array}$$

$$\begin{array}{c} \leq \mu P_{\text{1}}P_{\text{3}}\left(I_{AW}+I_{SW}\right)\left(\frac{\left(P_{\text{3}}P_{\text{4}}+k\sigma_{W}\left(\gamma_{W}+\mu \right)\right)}{\mu P_{\text{1}}P_{\text{3}}}-1\right)+\mu P_{\text{2}}P_{\text{5}}\left(I_{AR}+I_{SR}\right)\left(\frac{\left(P_{\text{5}}P_{\text{6}}+f\sigma_{R}\left(\gamma_{R}+\mu \right)\right)S}{\mu P_{\text{2}}P_{\text{5}}N}-1\right)\text{\textasciitilde{}}for\text{0.33em}\}S\leq N \end{array}$$

$$\begin{array}{c} \dot{F}=\mu P_{\text{1}}P_{\text{3}}\left(I_{AW}+I_{SW}\right)\left(R_{W}-1\right)+\mu P_{\text{2}}P_{\text{5}}\left(I_{AR}+I_{SR}\right)\left(R_{R}-1\right)\leq 0\text{\textasciitilde{}}for\text{0.33em}\}R_{\text{0}}\leq 1. \end{array}$$

Arising from the fact that the parameters of model are positive at all time as shown earlier, then F≤0 for $R_{\text{0}}\leq 1$ with F =0 given that $I_{AW}=I_{SW}=I_{AR}=I_{SR}=0$. Consequently, F is a Lyapunov function on $D_{\text{1}}$. Hence, using the invariance principle of Lasalle [47], with initial conditions in $D_{\text{1}}$, every equations in the treatment-free model (3), possess solutions that each approaches $\varepsilon_{\text{0}}ast\to \infty$.

Consequently, arising from Theorem 4, it has been established that the necessary and sufficient condition for the complete eradication of the disease from the given human population is the classical epidemiological requirement that the reproduction number of the disease be made less than unity.

#### 2.3.3 Existence and local stability of boundary equilibria.

The model (3) exhibits non-trivial equilibria, which occur when at least one of the infected variables is non-zero. However, these equilibria cannot be straightforwardly expressed in closed form. To investigate the existence and stability of these non-trivial equilibria, we adopt the approach outlined in [42]. Through careful examination, the potential equilibria of the model (3) are identified as follows:

1. Boundary equilibrium with only the wild strain (no resistant strain),

$\varepsilon_{W}$ = $\left(S^{*},E_{W}^{*},0,I_{AW}^{*},I_{SW}^{*},0,0\right)\text{.}$

2. Boundary equilibrium with only the drug-resistant strain (no wild strain),

$\varepsilon_{R}$ = $\left(S^{*},0,E_{R}^{*},0,0,I_{AR}^{*},I_{SR}^{*}\right)\text{.}$

3. Equilibria of co-existence of the two strain, $\varepsilon_{WR}=\left(S^{**},E_{W}^{**},E_{R}^{**},I_{AW}^{**},I_{SW}^{**},I_{AR}^{**},I_{SR}^{**}\right)\text{.}$

By solving the treatment-free model (3) at steady state, we obtain:


$$\begin{array}{c} S^{**}=\frac{\pi }{\left(\lambda_{W}^{**}+\lambda_{R}^{**}+\mu \right)},\text{\textasciitilde{}}E_{W}^{**}=\frac{\lambda_{W}^{**}\pi }{P_{\text{1}}\left(\lambda_{W}^{**}+\lambda_{R}^{**}+\mu \right)},\text{\textasciitilde{}}E_{R}^{**}=\frac{\lambda_{R}^{**}\pi }{P_{\text{2}}\left(\lambda_{W}^{**}+\lambda_{R}^{**}+\mu \right)},\text{\textasciitilde{}}I_{AW}^{**}=\frac{\lambda_{W}^{**}\pi \left(P_{\text{3}}P_{\text{4}}+\gamma_{W}k\sigma_{W}\right)}{\mu P_{\text{1}}P_{\text{3}}\left(\lambda_{W}^{**}+\lambda_{R}^{**}+\mu \right)}, \end{array}$$

$$\begin{array}{c} {I_{AW}^{**}=\frac{\lambda_{W}^{**}\pi \left(P_{\text{3}}P_{\text{4}}+\gamma_{W}k\sigma_{W}\right)}{\mu P_{\text{1}}P_{\text{3}}\left(\lambda_{W}^{**}+\lambda_{R}^{**}+\mu \right)},I}_{AR}^{**}=\frac{\lambda_{R}^{**}\pi f\sigma_{R}}{P_{\text{2}}P_{\text{5}}\left(\lambda_{W}^{**}+\lambda_{R}^{**}+\mu \right)},\text{\textasciitilde{}}I_{SR}^{**}=\frac{\lambda_{R}^{**}\pi \left(P_{\text{5}}P_{\text{6}}+\gamma_{R}f\sigma_{R}\right)}{\mu P_{\text{2}}P_{\text{5}}\left(\lambda_{W}^{**}+\lambda_{R}^{**}+\mu \right)}\text{.} \end{array}$$ (5)

Note that at the endemic state, we have:


$$\begin{array}{c} {\lambda_{W}^{**}=\frac{\beta_{W}\left(I_{AW}^{**}+I_{SW}^{**}\right)}{N^{**}}\text{\textasciitilde{}and\textasciitilde{}}\lambda }_{R}^{**}=\frac{\beta_{R}\left(I_{AR}^{**}+I_{SR}^{**}\right)}{N^{**}}\text{.} \end{array}$$ (6)

By putting the expressions in Eq (5) into (6), we obtain:


$$\begin{array}{c} \lambda_{W}^{**}=\phi_{\text{1}}\left(\lambda_{W}^{**},\lambda_{R}^{**}\right)=\frac{\frac{\beta_{W}\pi \lambda_{W}^{**}}{\left(\lambda_{W}^{**}+\lambda_{R}^{**}+\mu \right)}\left(\frac{k\sigma_{W}}{P_{\text{1}}P_{\text{3}}}+\frac{P_{\text{3}}P_{\text{4}}+\gamma_{W}k\sigma_{W}}{\mu P_{\text{1}}P_{\text{3}}}\right)}{N^{**}} \end{array}$$ (7)

$$\begin{array}{c} \lambda_{R}^{**}=\phi_{\text{2}}\left(\lambda_{W}^{**},\lambda_{R}^{**}\right)=\frac{\frac{\beta_{W}\pi \lambda_{R}^{**}}{\left(\lambda_{W}^{**}+\lambda_{R}^{**}+\mu \right)}\left(\frac{f\sigma_{R}}{P_{\text{2}}P_{\text{5}}}+\frac{P_{\text{5}}P_{\text{6}}+\gamma_{R}f\sigma_{R}}{\mu P_{\text{2}}P_{\text{5}}}\right)}{N^{**}} \end{array}$$

Where


$$\begin{array}{c} N^{**}=\frac{\pi }{\left(\lambda_{W}^{**}+\lambda_{R}^{**}+\mu \right)}\left[1+\lambda_{W}^{**}\left(\frac{\text{1}}{P_{\text{1}}}+\frac{k\sigma_{W}}{P_{\text{1}}P_{\text{3}}}+\frac{P_{\text{3}}P_{\text{4}}+\gamma_{W}k\sigma_{W}}{\mu P_{\text{1}}P_{\text{3}}}\right)+\lambda_{R}^{**}\left(\frac{\text{1}}{P_{\text{2}}}+\frac{f\sigma_{R}}{P_{\text{2}}P_{\text{5}}}+\frac{P_{\text{5}}P_{\text{6}}+\gamma_{R}f\sigma_{R}}{\mu P_{\text{2}}P_{\text{5}}}\right)\right] \end{array}$$

The equilibria of the model can be determined by identifying the fixed points of the system:


$$\begin{array}{c} x=\Phi \left(x\right)=\left(\begin{array}{c} \phi_{\text{1}}\left(\lambda_{W}^{**},\lambda_{R}^{**}\right)\\ \phi_{\text{2}}\left(\lambda_{W}^{**},\lambda_{R}^{**}\right) \end{array}\right)\text{,\textasciitilde{}where\textasciitilde{}}x=\left(\begin{array}{c} \lambda_{W}^{**}\\ \lambda_{R}^{**} \end{array}\right) \end{array}$$ (8)

as follows.

##### 2.3.3.1 Existence and stability of wild strain-only boundary equilibrium$\left(\varepsilon_{W}\right)$

The following is claimed.

**Theorem 2:** The treatment-free model (3) has a unique positive wild strain-only boundary equilibrium, $\varepsilon_{W}$, where $R_{R}<1<R_{W}.$ This equilibrium is locally asymptotically stable whenever it exists.

**Proof:** Let $R_{R}<1$, it can easily be shown that for $R_{R}<1$, $\left(I_{AR},I_{SR}\right)\to \left(0,0\right)$ as t→∞. In addition, it is clear from Eq (7) that $\phi_{\text{2}}\left(\lambda_{W}^{**},0\right)=0$. Thus, a fixed point of $\phi_{\text{1}}\left(\lambda_{W}^{**},\lambda_{R}^{**}\right)$ is obtained by solving the equation $\phi_{\text{1}}\left(\lambda_{W}^{**},0\right)=\lambda_{W}^{**}$. Hence, it follows that $\lambda_{W}^{**}$ is the root of the equation:


$$\begin{array}{c} \lambda_{W}^{**}\left(A_{\text{1}}\lambda_{W}^{**}+A_{\text{2}}\right)=0 \end{array}$$ (9)

Where $A_{\text{1}}=P_{\text{3}}\left(P_{\text{4}}+\mu \right)+k\sigma_{W}\left(\gamma_{W}+\mu \right)andA_{\text{2}}=\mu P_{\text{1}}P_{\text{3}}\left(1-R_{W}\right)$

Obviously, the roots of Eq (9) is $\lambda_{W}^{**}=0$ and $\lambda_{W}^{**}=\frac{-A_{\text{2}}}{A_{\text{1}}}$. The first solution$\lambda_{W}^{**}=0$, corresponding to the DFE$\varepsilon_{\text{0}}$. It can be shown that $A_{\text{1}}>0$ and $A_{\text{2}}>0(A_{\text{2}}<0)$ whenever $R_{W}<1\left(R_{W}>1\right)$. Hence, the solution $\lambda_{W}^{**}=\frac{-A_{\text{2}}}{A_{\text{1}}}>0$, whenever $A_{\text{2}}<0\left(R_{W}>1\right)$.

Furthermore, for the equilibrium $\varepsilon_{W}$ to exist alone, it is necessary that the resistant strain does not exist (that is,$R_{R}<1$). By combining all these we have that a unique wild strain-only boundary equilibrium $\left(\varepsilon_{W}\right)$exist whenever $R_{R}<1<R_{W}$.

Next, we explore the local stability property of $\varepsilon_{W}$, by taking note that the Jacobian of the system (8) is given by:


$$\begin{array}{c} J\left(\lambda_{W}^{**},\lambda_{R}^{**}\right)=\left(\begin{array}{cc} \frac{\partial \phi_{\text{1}}\left(\lambda_{W}^{**},\lambda_{R}^{**}\right)}{\partial \lambda_{W}^{**}} & \frac{\partial \phi_{\text{1}}\left(\lambda_{W}^{**},\lambda_{R}^{**}\right)}{\partial \lambda_{R}^{**}}\\ \frac{\partial \phi_{\text{2}}\left(\lambda_{W}^{**},\lambda_{R}^{**}\right)}{\partial \lambda_{W}^{**}} & \frac{\partial \phi_{\text{2}}\left(\lambda_{W}^{**},\lambda_{R}^{**}\right)}{\partial \lambda_{R}^{**}} \end{array}\right) \end{array}$$ (10)

So that


$$\begin{array}{c} \text{So\textasciitilde{}that\textasciitilde{}}J\left(\lambda_{W}^{**},0\right)=\left(\begin{array}{cc} \frac{\text{1}}{R_{W}} & {\frac{\partial \phi_{\text{1}}\left(\lambda_{W}^{**},\lambda_{R}^{**}\right)}{\partial \lambda_{R}^{**}}|}_{\left(\lambda_{W}^{**},0\right)}\\ 0 & \frac{R_{R}}{R_{W}} \end{array}\right)\text{.} \end{array}$$

We observe that the eigenvalues of $J\left(\lambda_{W}^{**},0\right)$ are $\lambda_{\text{1}}=\frac{\text{1}}{R_{W}}$ and $\lambda_{\text{2}}=\frac{R_{R}}{R_{W}}$. If $R_{R}<1$ in this case, then it implies that the wild strain-only equilibrium,$\varepsilon_{W}$, is locally asymptotically stable whenever $\left|\frac{\text{1}}{R_{W}}\right|<1\left(i.eR_{W}>1\right)$ and $\left|\frac{R_{R}}{R_{W}}\right|<1\left(i.eR_{R}<R_{W}\right)$. Hence,$\varepsilon_{W}$ is locally asymptotically stable whenever $R_{R}<1<R_{W}$.

##### 2.3.3.2 Existence and stability of resistant strain-only equilibrium $\left(\varepsilon_{R}\right)$

The following result is claimed.

**Theorem 3:** Whenever $R_{W}<1<R_{R}$, the treatment-free model (3) has a locally asymptotically stable unique resistant strain-only boundary equilibrium, $\varepsilon_{R}$.

**Proof:** Let $R_{W}<1$, so that $\left(I_{AW},I_{SW}\right)\to \left(0,0\right)$ as t→1. In addition, it is clear from Eq (7) that $\phi_{\text{1}}\left(0,\lambda_{R}^{**}\right)=0$. Therefore, by solving equation $\phi_{\text{2}}\left(0,\lambda_{R}^{**}\right)=\lambda_{R}^{**}$ we obtained a fixed point $\phi_{\text{2}}\left(\lambda_{W}^{**},\lambda_{R}^{**}\right)$. Consequently, we have the root of the equation:


$$\begin{array}{c} \lambda_{R}^{**}\left(A_{\text{3}}\lambda_{R}^{**}+A_{\text{4}}\right)=0 \end{array}$$ (11)

being $\lambda_{R}^{**}$.

With $A_{\text{3}}=P_{\text{5}}\left(P_{\text{6}}+\mu \right)+f\sigma_{R}\left(\gamma_{R}+\mu \right)andA_{\text{4}}=\mu P_{\text{2}}P_{\text{5}}\left(1-R_{R}\right)$

Obviously, the roots $\lambda_{R}^{**}=0$ and $\lambda_{R}^{**}=\left(\frac{-A_{\text{4}}}{A_{\text{3}}}\right)$ are the roots of Eq (11) corresponding to diseases free equilibrium. Whenever $R_{R}<1\left(R_{R}>1\right)$, it can be shown that $A_{\text{3}}>0$, while $A_{\text{4}}>0(A_{\text{4}}<0)$; therefore, whenever $A_{\text{4}}<0\left(R_{R}>1\right)$, then $\lambda_{R}^{**}=\frac{-A_{\text{4}}}{A_{\text{3}}}>0$. This is so since there is no wild strain $R_{W}<1$. Consequently, a unique resistant strain-only equilibrium,$\varepsilon_{R}$, exists whenever $R_{W}<1<R_{R}$.

By evaluating J at $\left(0,\lambda_{R}^{**}\right)$ we obtain:


$$\begin{array}{c} J\left(0,\lambda_{R}^{**}\right)=\left(\begin{array}{cc} \frac{R_{W}}{R_{R}} & 0\\ {\frac{\partial \phi_{\text{1}}\left(\lambda_{W}^{**},\lambda_{R}^{**}\right)}{\partial \lambda_{R}^{**}}|}_{\left(0,\lambda_{R}^{**}\right)} & \frac{\text{1}}{R_{R}} \end{array}\right) \end{array}$$

Here, we have that the requirement $\left|\frac{\text{1}}{R_{R}}\right|<1\left(i.eR_{R}>1\right)$ and $\left|\frac{R_{W}}{R_{R}}\right|<1\left(i.eR_{W}<R_{R}\right)$ for the local asymptotic stability of the equilibrium $\varepsilon_{R}$. Consequently, given that $R_{W}<1<R_{R}$, the drug resistant strain-only boundary equilibrium$\varepsilon_{R}$ is locally asymptotically stable.

#### 2.3.4 Existence and local stability of co-existence equilibria.

We rewrite the expression in Eq (7) as:


$$\begin{array}{c} \begin{array}{c} \lambda_{W}^{**}=\frac{\beta_{W}\left(I_{AW}^{**}+I_{SW}^{**}\right)}{N^{**}}\equiv \frac{\lambda_{W}^{**}R_{W}}{1+\lambda_{W}^{**}L_{\text{1}}+\lambda_{R}^{**}L_{\text{2}}}\\ \lambda_{R}^{**}=\frac{\beta_{R}\left(I_{AR}^{**}+I_{SR}^{**}\right)}{N^{**}}\equiv \frac{\lambda_{R}^{**}R_{R}}{1+\lambda_{W}^{**}L_{\text{1}}+\lambda_{R}^{**}L_{\text{2}}} \end{array} \end{array}$$ (12)

With $L_{\text{1}}=\frac{\text{1}}{P_{\text{1}}}+\frac{k\sigma_{W}}{P_{\text{1}}P_{\text{3}}}+\frac{P_{\text{3}}P_{\text{4}}+\gamma_{W}k\sigma_{W}}{\mu P_{\text{1}}P_{\text{3}}}$ and $L_{\text{2}}=\frac{\text{1}}{P_{\text{2}}}+\frac{f\sigma_{R}}{P_{\text{2}}P_{\text{5}}}+\frac{P_{\text{5}}P_{\text{6}}+\gamma_{R}f\sigma_{R}}{\mu P_{\text{2}}P_{\text{5}}}$.

Arising from Eq (12), consequently, we have:


$$\begin{array}{c} \begin{array}{c} \lambda_{W}^{**}L_{\text{1}}+\lambda_{R}^{**}L_{\text{2}}=R_{W}-1\\ \lambda_{W}^{**}L_{\text{1}}+\lambda_{R}^{**}L_{\text{2}}=R_{R}-1 \end{array} \end{array}$$ (13)

Due to the fact that Eq (13) has its left hand sides always positive, it is imperative that $R_{W}>1$ and $R_{R}>1$. The system (13)will be inconsistent given that $R_{W}\neq R_{R}$ such that in this case, there will be no positive co-existence equilibrium. Hence, condition for consistency of Eq (13) is that $R_{W}=R_{R}>1$. It is imperative to state that, in this circumstance, there will be infinitely many equilibria (otherwise called continuum endemic equilibria); it is worthy of note that this phenomenon also occurred in the work of Sharomi and Gumel [42] which centres on multi-strains model of HIV. Consequently, the implication is that we set $R_{W}=R_{R}=R>1$ to obtain:


$$\begin{array}{c} \lambda_{W}^{**}L_{\text{1}}+\lambda_{R}^{**}L_{\text{2}}=R-1 \end{array}$$ (14)

So that $0<\lambda_{W}^{**}<\frac{\left(R-1\right)}{L_{\text{1}}}$ and $0<\lambda_{R}^{**}<\frac{\left(R-1\right)}{L_{\text{2}}}$. We summarize the result as follows.

**Theorem 4:** There exist acontinuum of positive co-existence endemic equilibria, $\varepsilon_{WR}^{n}\left(n\in Z_{+}\right),$ for the treatment-free model (3) given that the following conditions are satisfied:


$$\begin{array}{c} \text{(a)\textasciitilde{}}R_{W}=R_{R}=R>1,\text{\textasciitilde{}(b)\textasciitilde{}}0<\lambda_{W}^{**}<\frac{\left(R-1\right)}{L_{\text{1}}}, \end{array}$$

$$\begin{array}{c} {\text{(c)\textasciitilde{}}0<\lambda_{R}^{**}<\frac{\left(R-1\right)}{L_{\text{2}}},\text{\textasciitilde{}(d)\textasciitilde{}}\lambda }_{W}^{**}=\frac{R-1-\lambda_{R}^{**}L_{\text{2}}}{L_{\text{1}}}, \end{array}$$

otherwise, there will be no continuum of positive co-existence endemic equilibria.

It is pertinent to state that for preservation of positivity of $\lambda_{W}^{**}$ and $\lambda_{R}^{**}$ in Eq (13), conditions (b)-(d) are needed.

Next we verify whether the continuum of positive co-existence endemic equilibria, $\varepsilon_{WR}^{n}$ is stable. We do this in the spirit of the work done by Sharomi and Gumel [42], by letting:


$$\begin{array}{c} R_{WR}^{n}=\frac{T_{\text{0}}+\sqrt{T_{\text{0}}^{\text{2}}-4T_{\text{1}}}}{\text{2}},\;n\in {\mathbb{Z}}_{+}. \end{array}$$

$$\begin{array}{c} \text{Where\textasciitilde{}}T_{\text{0}}={\left(\frac{\partial \phi_{\text{1}}}{\partial \lambda_{W}^{**}}+\frac{\partial \phi_{\text{2}}}{\partial \lambda_{R}^{**}}\right)|}_{\left(\lambda_{W}^{**},\lambda_{R}^{**}\right)}=\frac{1+R}{R},\text{\textasciitilde{}} \end{array}$$

$$\begin{array}{c} \text{and\textasciitilde{}}T_{\text{1}}={\left(\frac{\partial \phi_{\text{1}}}{\partial \lambda_{W}^{**}}\frac{\partial \phi_{\text{2}}}{\partial \lambda_{R}^{**}}-\frac{\partial \phi_{\text{1}}}{\partial \lambda_{R}^{**}}\frac{\partial \phi_{\text{2}}}{\partial \lambda_{W}^{**}}\right)|}_{\left(\lambda_{W}^{**},\lambda_{R}^{**}\right)}=\frac{\text{1}}{R}, \end{array}$$

with $R_{W}=R_{R}=R$. The following result is claimed.

**Theorem 5:** Given that R>1, there is neutrally stability for positive co-existence endemic equilibria $\varepsilon_{WR}^{n}$.

**Proof:** The eigenvalues of the Jacobian of Φ at $\lambda_{W}^{**}$ and $\lambda_{R}^{**}$ in the region (b) to (c), given by Eq (10), can be shown to satisfy characteristic polynomial:


$$\begin{array}{c} \chi^{\text{2}}-\chi {\left(\frac{\partial \phi_{\text{1}}}{\partial \lambda_{W}^{**}}+\frac{\partial \vartheta_{\text{2}}}{\partial \lambda_{R}^{**}}\right)|}_{\left(\lambda_{W}^{**},\lambda_{R}^{**}\right)}+{\left(\frac{\partial \phi_{\text{1}}}{\partial \lambda_{W}^{**}}\frac{\partial \phi_{\text{2}}}{\partial \lambda_{R}^{**}}-\frac{\partial \phi_{\text{1}}}{\partial \lambda_{R}^{**}}\frac{\partial \phi_{\text{2}}}{\partial \lambda_{W}^{**}}\right)|}_{\left(\lambda_{W}^{**},\lambda_{R}^{**}\right)}=0 \end{array}$$

Which is equivalent to:


$$\begin{array}{c} \chi^{\text{2}}-\chi \left(\frac{1+R}{R}\right)+\frac{\text{1}}{R}=0 \end{array}$$ (15)

The quadratic Eq (15) has its roots being χ=1 and $\chi =\frac{\text{1}}{R},$ hence, since χ=1 is an eigenvalue, then the family of positive co-existence endemic equilibria $\varepsilon_{WR}^{n}$ is neutrally stable.

Worthy of note is the fact that for the case *R = 1*, the trivial solution $\left(\lambda_{W}^{**},\lambda_{R}^{**}\right)=\left(0,0\right)$ is the solution of the system (13) is corresponding to the disease-free equilibrium.

We offer the following conjectures on competitive exclusion:

**CONJECTURE 1:** Whenever $R_{R}<R_{W}$ and $R_{W}<1$, there exist a unique, locally asymptotic stable positive wild strain-only boundary equilibrium, $\varepsilon_{W}$, for the treatment-free model (3).

Epidemiologically, the meaning of conjecture 1 is that for model without treatment (3), the dominant wild strain of the disease can be controlled when its reproduction number can be brought down to a value less than unity and the human population will not be invaded by the wild strain of the disease.

**CONJECTURE 2:** Whenever $R_{W}<R_{R}$ and $R_{R}<1$, there exist a unique, locally asymptotic stable positive resistant strain-only boundary equilibrium, $\varepsilon_{R}$, for the treatment-free model (3).

Likewise, the meaning of conjecture 2 is that for model without treatment (3), the dominant resistant strain can be controlled in the human population when its reproduction number can be brought down to a value less than unity and the population will not be invaded by the resistant strain of the disease.

### 2.4 Treatment model analysis

We investigate the treatment model (1) for its local and global asymptotic stability as follows.

#### 2.4.1 Local asymptotic stability.

We now consider the treatment model (1), its DFE is given by:


$$\begin{array}{c} \varepsilon_{\text{0}}^{t}=\left(S^{*},E_{W}^{*},E_{R}^{*},I_{AW}^{*},I_{SW}^{*},I_{AR}^{*},I_{SR}^{*},T^{*},R^{*}\right)=\left(\frac{\pi }{\mu },0,0,0,0,0,0,0,0\right) \end{array}$$ (16)

Likewise, as we did for the treatment-free model, the next generation matrices are given by:


$$\begin{array}{c} F=\left[\begin{array}{cccccc} 0 & \beta_{W} & \beta_{R} & 0 & 0 & 0\\ 0 & 0 & \beta_{W} & \beta_{R} & 0 & 0\\ 0 & 0 & 0 & 0 & 0 & 0\\ 0 & 0 & 0 & 0 & 0 & 0\\ 0 & 0 & 0 & 0 & 0 & 0\\ 0 & 0 & 0 & 0 & 0 & 0 \end{array}\right]\text{\textasciitilde{}}V=\left[\begin{array}{cccccc} B_{\text{1}} & 0 & 0 & 0 & 0 & 0\\ 0 & B_{\text{2}} & 0 & 0 & 0 & 0\\ -k\sigma_{W} & 0 & B_{\text{3}} & 0 & 0 & 0\\ \beta_{\text{4}} & 0 & -\gamma_{W} & B_{\text{5}} & 0 & 0\\ 0 & -f\sigma_{R} & 0 & 0 & B_{\text{6}} & 0\\ 0 & -\beta_{\text{7}} & 0 & 0 & -\gamma_{R} & B_{\text{8}} \end{array}\right] \end{array}$$

Where $B_{\text{1}}=\sigma_{W}+\mu$, $B_{\text{2}}=\left(\sigma_{R}+\mu \right)$,$B_{\text{3}}=\gamma_{W}+\mu$, $B_{\text{4}}=\left(1-k\right)\sigma_{W}$,

$B_{\text{5}}=\alpha_{W}+\mu$, $B_{\text{6}}=\gamma_{R}+\mu$,$B_{\text{7}}=\left(1-f\right)\sigma_{R}$ and $B_{\text{8}}=\alpha_{R}+\mu$.

Consequently, the reproduction number for the treatment model, denoted by $R_{\text{0}}^{t}=\rho \left(FV^{-1}\right)$, is given as:


$$\begin{array}{c} R_{\text{0}}^{t}=max\left\{R_{W}^{t},R_{R}^{t}\right\} \end{array}$$

With $R_{W}^{t}=\frac{\beta_{W}\left(\left(\gamma_{W}+\mu \right)\left(1-k\right)\sigma_{W}+\left(\alpha_{W}+\mu \right)k\sigma_{W}+\gamma_{W}k\sigma_{W}\right)}{\left(\sigma_{W}+\mu \right)\left(\gamma_{W}+\mu \right)\left(\alpha_{W}+\mu \right)}\text{.}$

And $R_{R}^{t}=\frac{\beta_{R}\left(\left(1-f\right)\sigma_{R}\left(\alpha_{R}q\varphi +\left(\gamma_{R}+\mu \right)\left(\varepsilon +\varphi +\mu \right)\right)+f\sigma_{R}\left(\left(\varepsilon +\varphi +\mu \right)\left(\left(\alpha_{R}+\mu \right)+\gamma_{R}\right)-\left(1-q\right)\varphi \alpha_{R}\right)\right)}{\left(\sigma_{R}+\mu \right)\left(\left(\gamma_{R}+\mu \right)\left(\alpha_{R}+\mu \right)\left(\varepsilon +\varphi +\mu \right)-\alpha_{R}\gamma_{R}q\varphi -\alpha_{R}\left(\gamma_{R}+\mu \right)\left(1-q\right)\varphi \right)}\text{.}$

By theorem 2 of van den Driessche [41], the following result holds.

**Lemma 3:** For the treatment model (1), its disease free equilibrium given by Eq (16) is locally asymptotically stable if $R_{\text{0}}^{t}<1$, and wouldn’t be stable if $R_{\text{0}}^{t}>1$.

The implication of the above lemmaepidemiologically is that the use of antibiotics such as Azithromycin and Moxifloxacinfor the treatment of the disease can lead to the elimination of *M. Genitalium* from the given human population when the epidemiological threshold $R_{\text{0}}^{t}$ is not up to unity (that is $R_{\text{0}}^{t}<1$) if the initial sizes of the subpopulations of the model are in the basin of attraction of $\varepsilon_{\text{0}}^{t}$. To ensure that the use of these afore-mentioned antibiotics as treatment regimen for the disease will lead to the elimination of both strains of the disease regardless of the initial sizes of the subpopulations of the model, it becomes pertinent to show that the DFE of the model (1) is globally asymptotically stable. This is what we explore in the section below.

#### 2.4.2 Global stability of disease-free equilibrium.

**Theorem 6:** For the treatment model (1), its disease free equilibrium given by Eq (16) is locally asymptotically stable in the domain D given that $R_{\text{0}}^{t}<1$.

**Proof:** We prove this theorem using the comparison theorem technique; by first rewriting in compact form, the infected components in the treatment model (1) to obtain:


$$\begin{array}{c} \left(\begin{array}{c} \frac{dE_{W}\left(t\right)}{dt}\\ \frac{dE_{R}\left(t\right)}{dt}\\ \frac{dI_{AW}\left(t\right)}{dt}\\ \frac{dI_{SW}\left(t\right)}{dt}\\ \frac{dI_{AR}\left(t\right)}{dt}\\ \frac{dI_{SR}\left(t\right)}{dt}\\ \frac{dT\left(t\right)}{dt}\\ \frac{dR\left(t\right)}{dt} \end{array}\right)=\left(F-V\right)\left(\begin{array}{c} E_{W}\left(t\right)\\ E_{R}\left(t\right)\\ I_{AW}\left(t\right)\\ I_{SW}\left(t\right)\\ I_{AR}\left(t\right)\\ I_{SR}\left(t\right)\\ T\left(t\right)\\ R\left(t\right) \end{array}\right)-\left(1-\frac{S}{N}\right)\left(\begin{array}{cccccccc} 0 & 0 & \beta_{W} & \beta_{W} & 0 & 0 & 0 & 0\\ 0 & 0 & 0 & 0 & \beta_{R} & \beta_{R} & 0 & 0\\ 0 & 0 & 0 & 0 & 0 & 0 & 0 & 0\\ 0 & 0 & 0 & 0 & 0 & 0 & 0 & 0\\ 0 & 0 & 0 & 0 & 0 & 0 & 0 & 0\\ 0 & 0 & 0 & 0 & 0 & 0 & 0 & 0\\ 0 & 0 & 0 & 0 & 0 & 0 & 0 & 0\\ 0 & 0 & 0 & 0 & 0 & 0 & 0 & 0 \end{array}\right)\left(\begin{array}{c} E_{W}\left(t\right)\\ E_{R}\left(t\right)\\ I_{AW}\left(t\right)\\ I_{SW}\left(t\right)\\ I_{AR}\left(t\right)\\ I_{SR}\left(t\right)\\ T\left(t\right)\\ R\left(t\right) \end{array}\right) \end{array}$$

With F and V matrices whose interpretation has been given in the preceding section. Since S(t)≤N(t) (for all t≥0) in D, then,


$$\begin{array}{c} \left(\begin{array}{c} \frac{dE_{W}\left(t\right)}{dt}\\ \frac{dE_{R}\left(t\right)}{dt}\\ \frac{dI_{AW}\left(t\right)}{dt}\\ \frac{dI_{SW}\left(t\right)}{dt}\\ \frac{dI_{AR}\left(t\right)}{dt}\\ \frac{dI_{SR}\left(t\right)}{dt}\\ \frac{dT\left(t\right)}{dt}\\ \frac{dR\left(t\right)}{dt} \end{array}\right)\leq \left(F-V\right)\left(\begin{array}{c} E_{W}\left(t\right)\\ E_{R}\left(t\right)\\ I_{AW}\left(t\right)\\ I_{SW}\left(t\right)\\ I_{AR}\left(t\right)\\ I_{SR}\left(t\right)\\ T\left(t\right)\\ R\left(t\right) \end{array}\right) \end{array}$$ (17)

Since matrix F−V has its eigenvalues being negative real parts, whenever $R_{\text{0}}^{t}<1$, the implication is that the linearized differential inequality system (17) is stable.

Hence, $\left(E_{W}\left(t\right),E_{R}\left(t\right),I_{AW}\left(t\right),I_{SW}\left(t\right),I_{AR}\left(t\right),I_{SR}\left(t\right),T\left(t\right),R\left(t\right)\right)\to \left(0,0,0,0,0,0,0,0\right)$ as t→∞. By putting $E_{W}+E_{R}+I_{AW}+I_{SW}+I_{AR}+I_{SR}+T+R=0$ into the first expression of Eq (1) we obtain $S\left(t\right)\to \frac{\pi }{\mu }$ as t→∞.

Therefore, $\left(S\left(t\right),E_{W}\left(t\right),E_{R}\left(t\right),I_{AW}\left(t\right),I_{SW}\left(t\right),I_{AR}\left(t\right),I_{SR}\left(t\right),T\left(t\right),R\left(t\right)\right)\to \left(\frac{\pi }{\mu },0,0,0,0,0,0,0,0\right)$

as t→∞ for $R_{\text{0}}^{t}<1$, so that $\varepsilon_{\text{0}}^{t}$ is globally asymptotically stable in domain D if $R_{\text{0}}^{t}<1$.

Epidemiologically, theorem 6 aboveimplies that if the adoption of the treatment of the disease with antibiotics can aid the reduction of the maximum of the reproduction numbers of the two strains such that their values are not up to unity, then this will ultimately result to elimination of both strains of the disease from the community.

## 3 Sensitivity analysis for the model

Determining the top rank parameters that is of greater influence on the disease transmission dynamics is pertinent, hence we embark on sensitivity analysis of the model so as to obtain parameters that must be diligently targeted towards bringing down the reproduction number of the disease to a value that is not up to unity [48,49]. We did this for each of the two strains of the disease so as to obtain the determining factors for eradication of the two strains of the disease and ultimately, the disease from the population under reference.

**Fig 2:**
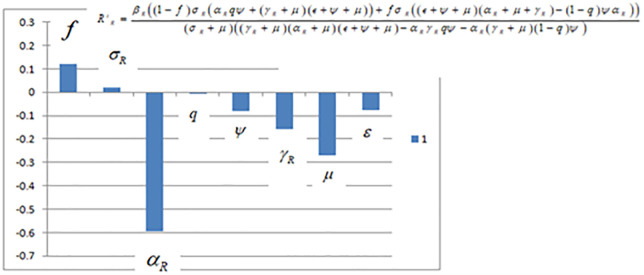
[34] Schematic diagram of the sensitivity indices for resistant strain of thetreatment model (1).

Table 2 presents the Partial Rank Correlation Coefficient (PRCC) values for the parameters associated with the resistant strain in the treatment model (1). PRCC is a sensitivity analysis method that evaluates the strength and direction of the relationship between input parameters and the model's output (response function) while accounting for non-linear and non-monotonic relationships [40,50]. Fig 2 is a schematic diagram of sensitivity indices which highlights the key parameters influencing drug-resistant strains, aiding in understanding, optimizing treatments, and prioritizing interventions [40].

**Table 2:** PRCC values of the parameters of resistant strain of the treatment model (1), with $R_{R}$ as the output (response function).

S/N	Parameters	PRCC ($R_{R}$)
1.	$\beta_{R}$	1
2.	f	0.1698
3.	$\sigma_{R}$	0.0204
4.	$\alpha_{R}$	-0.5948
5.	q	-0.0045
6.	$\psi_{R}$	-0.0795
7.	$\gamma_{R}$	-0.1592
8.	μ	-0.2694
9.	ε	-0.0765

From the sensitivity analysis conducted for resistant strain of the treatment model (1), it could be seen that the dominant parameters that drive the dynamic of the spread of the resistance strain of the disease are fraction of individuals that resist treatment(*q)*, rate of recovery of treated individuals (ε), progression rate from exposed to infected class for resistance strain ($\sigma_{R}$), and progression rate from infected asymptomatic to symptomatic resistant strain ($\gamma_{R}$). These parameters are those that will bring down the reproduction number of the resistant strain of the disease significantly when their values are brought down to their minimum, hence policy makers must carefully target them with a view to doing all what can be done to keep their values at the lowest at all times. By doing this, the reproduction number of the resistant strain of the disease will be significantly brought down to a value less than unity and this strain of the disease will fail to invade the population, hence it will ultimately die out in no time.

**Fig 3:**
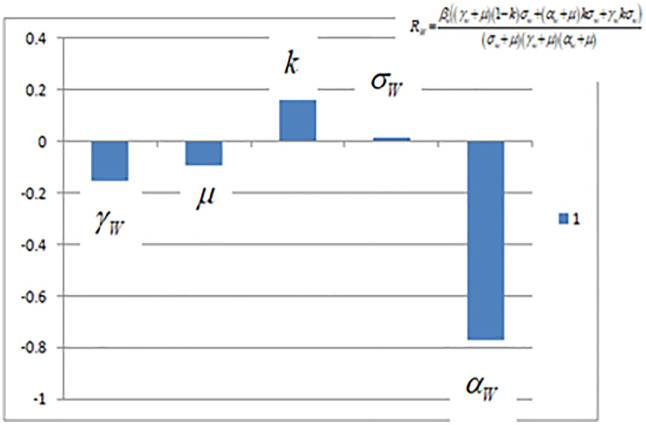
Schematic diagram of the sensitivity indices for wild strain of the treatment model (1).

Table 3 provides the Partial Rank Correlation Coefficient (PRCC) values for the parameters associated with the wild strain in the treatment model. PRCC is a statistical measure used in sensitivity analysis to determine how variations in model parameters influence the output (response function), while accounting for potential non-linear relationships [34,51]. Fig 3 is a schematic diagram of sensitivity indices for the wild strain highlights key parameters influencing its dynamics, aiding in optimizing treatment and preventing resistance [34].

**Table 3:** PRCC values of the parameters of Wild strain of the treatment model (1), with $R_{W}$ as the output (response function).

S/N	Parameters	PRCC ($R_{W}$)
1.	$\beta_{W}$	1
2.	$\sigma_{W}$	0.0171
3.	u	-0.0919
4.	$\alpha_{R}$	-0.7725
5.	$\gamma_{W}$	-0.1526
6.	*K*	0.1597

## 4 Numerical simulation

Using MATLAB programming language, we carried out numerical simulations of the epidemiological model so formulated as to be able to make observation as regards the dynamics of *M. Genitalium* model over time. In doing this, we adopted parameters in Table 2 which are derived from literature. The main goal of this numerical simulations is the validation and illustration of some of the theoretical results obtained earlier.

Table 4 outlines the parameter values used in the model, providing a comprehensive reference for the constants and variables that define the system's behavior [34,40]. Each parameter is assigned a specific value, ensuring consistency and reproducibility in the model's simulations and analysis.

**Table 4:** Parameter values for the model.

𝐏𝐚𝐫𝐚𝐦𝐞𝐭𝐞𝐫𝐬	𝐕𝐚𝐥𝐮𝐞𝐬	Sources
π	1000	[42]
$\beta_{W}$	0.816	[38]
$\beta_{R}$	0.816	[38]
$\sigma_{W}$	1.8	Assumed
$\sigma_{R}$	1.5	Assumed
$\gamma_{W}$	0.67	Inferred from [42]
$\gamma_{R}$	0.33	Inferred from [42]
f	0.71	Assumed
$\alpha_{W}$	0.43	Inferred from [42]
$\alpha_{R}$	0.12	Inferred from [42]
ε	0.8	[38]
φ	0.12	[38]
q	0.18	Assumed
μ	1/32	[42]
ρ	0.71	Assumed
k	0.31	Inferred from [38]
N	1008	[38]

The parameter values used in the model are justified as follows: The progression rates from exposure to infectiousness for both the wild and resistant strains were set at $\sigma_{W}=1.8$ and $\sigma_{R}=1.5$, respectively. These values are based on the reported incubation periods for Mycoplasma genitalium, which range from 0.5 to 0.8 weeks (3–6 days). Taking the midpoint of 4 days gives a progression rate of $1/4\approx 0.25d{\}}^{\text{-1}}$, which when converted to weeks gives $0.25\times 7\approx 1.75wk{\}}^{\text{-1}}$. The values used in the model, 1.8 for the wild strain and 1.5 for the resistant strain, reflect the assumption that resistant infections tend to progress more slowly to symptomatic stages. The fraction of asymptomatic infections, f=0.71, was selected based on meta-analysis data, which reports asymptomatic rates ranging from 60% to 80%. The mid-range value of 0.71 was chosen to reflect the observed prevalence of asymptomatic infections in the UK surveillance study referenced in [42]. The failure probability for treatment, q=0.18, was assumed based on meta-analyses, which suggest a 15%–20% failure rate for macrolide treatments. The relative fitness of the resistant strain, ρ=0.71, was based on in-vitro studies which indicate that the macrolide-resistant strain replicates at approximately 70% of the wild-type rate when antibiotics are not present. The progression rates from asymptomatic to symptomatic infection for both the wild and resistant strains, $\gamma_{W}=0.67$ and $\gamma_{R}=0.33$, were inferred from data in [42], which reports average progression times of 1.5 weeks for the wild strain and 3 weeks for the resistant strain. The treatment-seeking rates, $\alpha_{W}=0.43$ and $\alpha_{R}=0.12$, were inferred from the time to treatment-seeking behavior, with wild-type individuals presenting more quickly than resistant ones. The loss-of-immunity rate, k=0.31, was inferred from the duration of post-infection immunity, which was approximately 3.2 weeks, as reported in [38]. Finally, natural mortality, μ, was taken from national demographic data reported in [42] as $0.000039wk{\}}^{\text{-1}}$, which was used in the model simulations.

**Fig 4:**
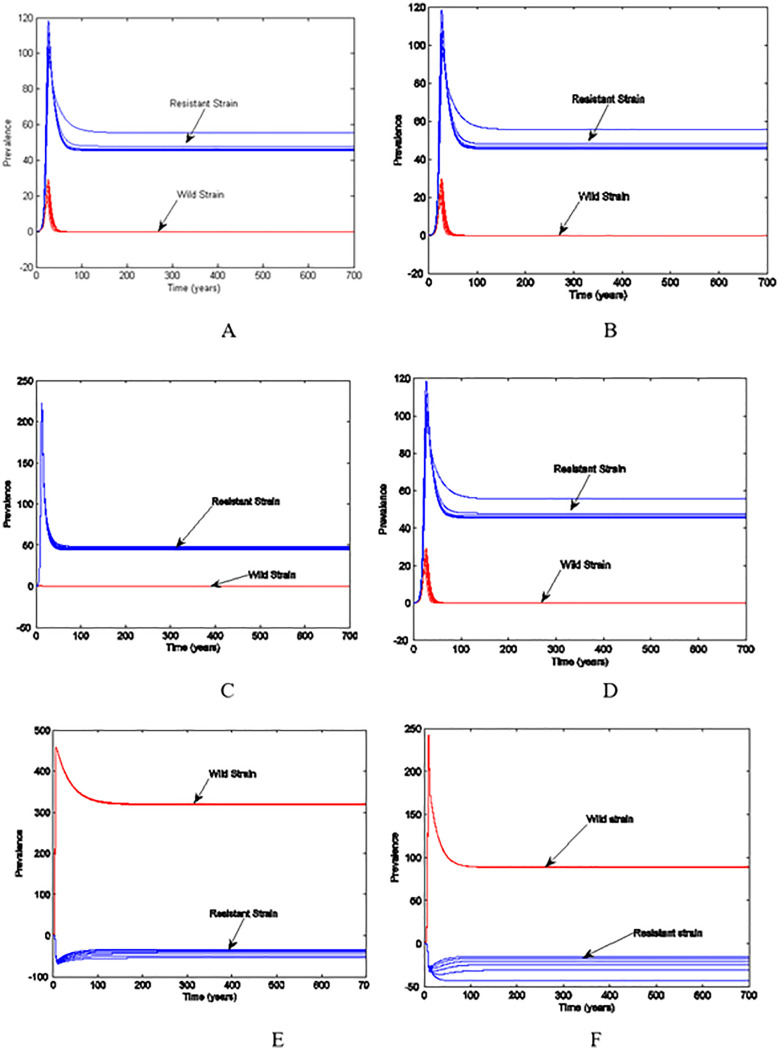
Prevalence given by total number of infected individuals divided by the total population as a function of time (years) for treatment-free model (3). 4(a) $\beta_{W}=\beta_{R}=0.5$, $R_{W}=0.2588$ & $R_{R}=0.0485$; 4(b) $R_{W}>R_{R}>1$, $\beta_{W}=0.5,\beta_{R}=0.3$. 4(c) $R_{R}>R_{W}>1$, $\beta_{W}=0.1,\beta_{R}=0.7$; 4(d). $R_{W}>R_{R}>1$$\beta_{W}=4.6,\beta_{R}=10.9$. 4(e) $R_{R}=R_{W}>1$, 4(f) $R_{R}=R_{W}>1$, $\beta_{W}=3.9,\beta_{R}=20.9$.

**Treatment-free model:** We first simulated the model without treatment, with $\beta_{W}=\beta_{R}=0.5$ and other parameter values as given in Table 2, where we obtained the reproduction number for the wild (drug-resistant) strain of the disease being $R_{W}=0.2588]([R_{R}=0.04850$), so that $R_{\text{0}}=0.2588<1$. This is a confirmation of Theorem 1, that the disease-free equilibrium of the model is globally asymptotically stable. The simulation of the model under this scenario, with various initial condition is depicted by Fig 4A, this confirms the global asymptotic stability property of the model as initially proven qualitatively in Sect 3.2. This finding aligns with real-world observations where M. genitalium infections cannot persist in populations without sufficient transmission rates, corresponding to reproduction numbers below 1. The parameter values used are derived from epidemiological studies of M. genitalium (see Table 2), ensuring biological relevance.

Additional simulations of the model (3) were carried out for Ri>Rj, with $R_{i}>1$ where (i,j=W,R,,and,i≠j), these simulations are as shown in Fig 4B, Fig 4C and Fig 4D where it can be observed that the strain with higher reproduction number drives out the other, a phenomenon commonly refers to as competitive exclusion. Note that this is a confirmation and validity of theorems 2 and 3 and conjectures 1 and 2. This competitive exclusion dynamic mirrors clinical observations in settings where antibiotic resistance has emerged; specifically, the model captures the pattern documented in surveillance studies where increased macrolide resistance in M. genitalium has been observed following widespread azithromycin use [44,45]. The predominance of resistant strains in high-treatment settings validates our model's prediction of strain replacement under selection pressure.

However, observe that in Fig 4E and Fig 4F, when the reproduction number of the resistant strain $R_{R}$ is equals to that of wild strain for $R_{W}$ is greater than one, that is, $R_{R}=R_{W}>1$ then the resistant strain which is the one with larger reproduction number drives the other one, wild strain to extinction. Though this equal reproduction number scenario is theoretical, it helps validate the mathematical robustness of our model and provides insights into potential transitional states during the evolution of resistance. The model's behavior under these conditions agrees with ecological competition principles observed in similar pathogen dynamics.

**Fig 5:**
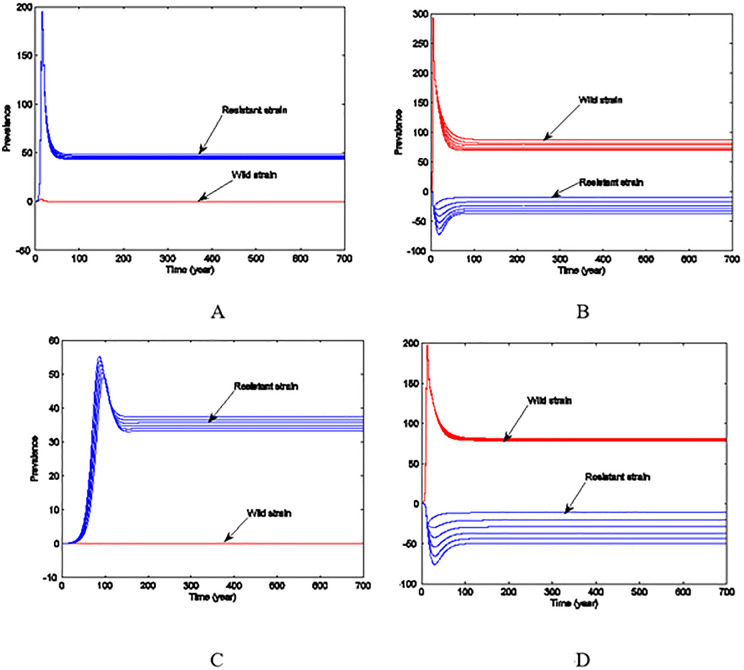
Prevalence given by total number of infected individuals divided by the total population as a function of time (years) for treatment model (1). 4(a) $R_{W}^{t}<1<R_{R}^{t}$, $\beta_{W}=0.9,\beta_{R}=1.4$; 4(b) $R_{R}^{t}<1<R_{W}^{t}$, $\beta_{W}=3.9,\beta_{R}=0.4$; 4(c) $R_{W}^{t}<R_{R}^{t}<1$,$\beta_{W}=0.4,\beta_{R}=0.4$; 4(d) $R_{R}^{t}<R_{W}^{t}<1$; $\beta_{W}=1.7,\beta_{R}=0.4$.

**Treatment model:** We simulated the treatment model (1) next. By using the parameters in Table 2, as can be seen from Fig 5A and Fig 5B for $R_{W}^{t}<1<R_{R}^{t}$ and $R_{R}^{t}<1<R_{W}^{t}$ respectively, there is co-existence of both strains in the community with the system converging to low-endemicity steady state. From Fig 5C and Fig 5D, it could be observed that the strain with higher reproduction number drives out the other giving rise to competitive exclusion in line with theorem 2 and 3 and conjectures 1 and 2. These coexistence and competitive exclusion patterns align with clinical observations from longitudinal studies in different geographical regions. For instance, the coexistence scenario in Fig 5A and Fig 5B resembles the epidemiological situation in regions with moderate treatment coverage, where both wild-type and resistant strains circulate simultaneously [52]. The strain replacement shown in Fig 5C and Fig 5D corresponds to high-treatment settings such as in Australia and some European countries, where macrolide-resistant *M. genitalium* has become predominant, reaching prevalence rates of 40–80% [53,54].

**Fig 6:**
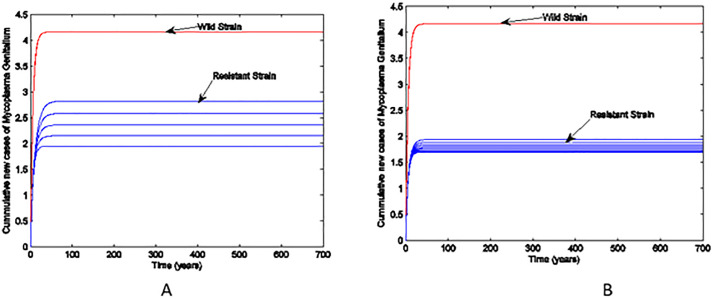
Cumulative number of new cases of Mycoplasma Genitalium as a function of time (in years) for treatment model. *6(a)* $R_{R}^{t}<R_{W}^{t}<1$*- varying q (f*raction of individuals that resist treatment); *6(b)* $R_{R}^{t}<R_{W}^{t}<1$- varyingε (Rate of recovery of treated individuals).

We plotted cumulative number of new cases of *M. Genitalium* as a function of time (in years) for treatment model while varying fraction of individuals that resist treatment as shown Fig 6A, it could be seen that, expectedly, this has a significant effect on the cumulative number of new cases of individual infected with resistant strain of the disease which increases as its number increases. While varying fraction of individuals that resist treatment has no effect on cumulative number of new infections for wild strain. This finding corresponds with real-world epidemiological data showing increased cumulative burden of resistant infections in settings with higher treatment failure rates. Our model predictions parallel surveillance data from the UK, Japan, and Australia, where increasing resistance rates have been temporally associated with higher overall disease burden [55].

On the other hand from Fig 6B, rate of recovery of treated individuals when varied for the treatment model, while it has significant effect on the drug-resistant strain of the disease, it is of no significant effect on the wild strain of the disease. This differential impact on wild versus resistant strains validates against clinical observations where improved treatment efficacy for susceptible infections has minimal impact on resistant strain prevalence once established in a population. The asymptotic behavior shown in Fig 6(b) matches the plateau effect observed in longitudinal studies where increased treatment intensity beyond certain thresholds yields diminishing returns for overall disease control [46].

To further validate our model against real-world data, we compared our equilibrium prevalence predictions with reported M. genitalium prevalence rates from systematic reviews and meta-analyses. The equilibrium prevalence ranges of 30–55% for resistant strains and 0–5% for wild strains under high treatment scenarios (Fig 5) align with epidemiological data from high-income countries with established resistance problems, such as Denmark and Australia, where resistant M. genitalium comprises 40–70% of cases [56]. Similarly, our predictions for settings with lower treatment intensity (early phase curves in Fig 5) reflect the epidemiological profile of regions with emerging resistance, where wild-type strains still predominate but resistant strains are increasing.The time scales predicted by our model for resistance emergence and stabilization (approximately 100–200 years to reach equilibrium) may appear extended compared to clinical observations of more rapid resistance emergence. However, this reflects the population-level perspective of our model versus the more rapidly observable clinical resistance patterns in treated individuals. When considering appropriate time scaling factors and accounting for heterogeneity in sexual networks not explicitly modeled here, our predicted temporal dynamics provide reasonable approximations of observed resistance patterns.

## 5 Conclusion

In this work, formulation of a deterministic epidemiological model was done by incorporating a two-strain (drug-sensitive and drug-resistant strain) to capture the system of non-linear differential equations governing the transmission dynamics of Mycoplasma Genitalium so as to have a sound understanding of how the disease transmission can be effectively controlled with the use of antibiotics for its treatment. Analyzing the treatment-free model and the model with treatment qualitatively and quantitatively showed that there exists a global asymptotically stable disease-free equilibrium when the greater of the two reproduction numbers for each of the models was not up to unity; this is an implication of the feasibility of the effective control or complete eradication of the disease by treatment of infected individuals with antibiotics, such that this treatment can make the greater of the reproduction numbers not up to unity. A major revelation from the study is that for the model without treatment, the strain with the higher reproduction number is always dominant over the other at steady state while the other strain dies out in the population. For the treatment model, there is occurrence of competitive exclusion where the strain with the larger reproduction number drives the other to extinction only when $R_{R}^{t}>1$and $R_{R}^{t}>R_{W}^{t}$.

Furthermore, it is revealed through this study that consistent use of treatment of the disease with antibiotics despite the risk of the emergence and transmission of the drug-resistant strain of the disease, can ultimately and effectively checkmate the widespread disease burden or even lead to its complete elimination when certain conditions are met. From the sensitivity analysis carried out for the model, it was discovered that the top-ranked parameters that greatly influenced the spread of the drug-resistant strain of the disease in the population under study are the fraction of individuals that resist treatment (q), the rate of recovery of treated individuals (ε), progression rate from exposed to infected class with the resistant strain ($\sigma_{R}$), and progression rate from infected asymptomatic class to symptomatically infected with resistant strain ($\gamma_{R}$). The implication of this is that the policymakers in the healthcare sector must target these aforementioned parameters carefully so that their values are brought down to their lowest, such that the reproduction number of the strain is made not to be up to unity in value, as a prerequisite for effective control of this drug-resistant strain of the disease. Likewise, from the sensitivity analysis, the discovery is that the top-ranked parameters that greatly influenced the spread of the other strain of the disease (the wild strain) are the natural death rate (u), progression rate from class of infected asymptomatic to that of symptomatic individuals with resistant strain ($\gamma_{W}$), and progression from the compartment of infected with resistance strain to the Treated compartment ($\alpha_{R}$). Epidemiologically, it is recommended that the policymakers in the healthcare sector must do all they can to ensure that these top-ranked parameters are well-targeted so that their values are brought down to their lowest for the reproduction number of the disease not to be up to unity, which will lead to the two strains of the disease being controlled effectively in the population. By so doing, the spread of the disease will ultimately be brought under control effectively.

Another major recommendation from this study is that further study be focused on the discovery and use of alternative drugs and other treatment regimens that can be of valuable help to combat the spread of the drug-resistant strain of the disease.

As an area of further contribution to knowledge, this work can be improved upon by incorporating time-dependent control measures into the model and analyzing it with a view to procuring optimal control measures that will help in the effective combat of the spread of the disease and obtaining cost-effective ways of mitigating the disease burden. Another area of further contribution to knowledge is by reformulating the model as a Caputo-based fractional order derivative model or Atangana-Baleanu-based fractional order model and using appropriate numerical schemes to provide a solution to the resulting system of non-linear fractional order differential equations.

Furthermore, it is crucial to emphasize the importance of robust surveillance systems and effective antibiotic stewardship programs. These strategies complement mathematical modeling and treatment policies by providing essential data for monitoring disease spread, guiding timely interventions, and mitigating the development of antibiotic resistance.

## Supporting information

S1 File MATLAB code and guide. (DOCX)
