# Supplementary material for: Mathematical analysis of Mycoplasma Genitalium transmission dynamics: A compartmental model with nonlinear interactions and treatment effects
Source: PLOS Glob Public Health. 2026 Jul 27;6(7):e0006834. doi: 10.1371/journal.pgph.0006834 (PMC13405295; doi:10.1371/journal.pgph.0006834)
Supplement: S1 File — (DOCX) [file pgph.0006834.s001.docx]

# Mathematical Analysis of *Mycoplasma Genitalium* Transmission Dynamics: A Compartmental Model with Nonlinear Interactions and Treatment Effects

# MATLABCODES AND GUIDE FOR THE ABOVE MANUSCRIPT TILTLE

clc; clear; close all;

%% ======================

% PARAMETERS (Table 4)

%% ======================

Lambda = 10; % recruitment rate

beta_w = 0.8; % transmission (wild)

beta_r = 0.6; % transmission (resistant)

sigma_w = 1.8; % exposed -> infectious (wild)

sigma_r = 1.5; % exposed -> infectious (resistant)

p_w = 0.71; % fraction asymptomatic (wild)

p_r = 0.71; % fraction asymptomatic (resistant)

gamma_w = 0.67; % asymptomatic -> symptomatic (wild)

gamma_r = 0.33; % asymptomatic -> symptomatic (resistant)

tau_w = 0.5; % treatment rate (wild)

tau_r = 0.4; % treatment rate (resistant)

delta = 0.3; % recovery rate

q = 0.2; % treatment resistance fraction

mu = 0.02; % natural death

omega = 0.1; % immunity loss

params = [Lambda beta_w beta_r sigma_w sigma_r p_w p_r ...

gamma_w gamma_r tau_w tau_r delta q mu omega];

%% ======================

% INITIAL CONDITIONS

%% ======================

S0 = 1000;

Ew0 = 10;

Er0 = 5;

Iaw0 = 5;

Isw0 = 5;

Iar0 = 3;

Isr0 = 3;

T0 = 2;

R0 = 0;

X0 = [S0 Ew0 Er0 Iaw0 Isw0 Iar0 Isr0 T0 R0];

tspan = [0 200];

%% ======================

% SOLVE SYSTEM

%% ======================

[t,X] = ode45(@(t,X) model1(t,X,params), tspan, X0);

%% ======================

% FIGURE 4 (a–f)

%% ======================

figure;

subplot(2,3,1); plot(t,X(:,1)); title('S');

subplot(2,3,2); plot(t,X(:,2)); title('E_w');

subplot(2,3,3); plot(t,X(:,3)); title('E_r');

subplot(2,3,4); plot(t,X(:,4)); title('I_aw');

subplot(2,3,5); plot(t,X(:,5)); title('I_sw');

subplot(2,3,6); plot(t,X(:,6)); title('I_ar');

%% ======================

% FIGURE 5 (a–d)

%% ======================

figure;

subplot(2,2,1); plot(t,X(:,7)); title('I_sr');

subplot(2,2,2); plot(t,X(:,8)); title('Treated');

subplot(2,2,3); plot(t,X(:,9)); title('Recovered');

subplot(2,2,4); plot(t,sum(X,2)); title('Total Population');

%% ======================

% FIGURE 6 (a–b) PARAMETER SENSITIVITY

%% ======================

% Example: vary treatment rate

tau_values = [0.2 0.5 0.8];

figure;

hold on;

for i = 1:length(tau_values)

params(10) = tau_values(i); % tau_w

[t,X] = ode45(@(t,X) model1(t,X,params), tspan, X0);

plot(t,X(:,5)); % symptomatic wild

end

legend('0.2','0.5','0.8');

title('Effect of Treatment Rate on I_{sw}');

figure;

hold on;

for i = 1:length(tau_values)

params(11) = tau_values(i); % tau_r

[t,X] = ode45(@(t,X) model1(t,X,params), tspan, X0);

plot(t,X(:,7)); % symptomatic resistant

end

legend('0.2','0.5','0.8');

title('Effect on Resistant Infection');

**MODEL FUNCTION FILE**

Create a separate file: **model1.m**

function dXdt = model1(~,X,p)

% Unpack variables

S = X(1);

Ew = X(2);

Er = X(3);

Iaw = X(4);

Isw = X(5);

Iar = X(6);

Isr = X(7);

T = X(8);

R = X(9);

% Parameters

Lambda = p(1);

beta_w = p(2);

beta_r = p(3);

sigma_w = p(4);

sigma_r = p(5);

p_w = p(6);

p_r = p(7);

gamma_w = p(8);

gamma_r = p(9);

tau_w = p(10);

tau_r = p(11);

delta = p(12);

q = p(13);

mu = p(14);

omega = p(15);

N = sum(X);

% Force of infection

lambda_w = beta_w*(Iaw + Isw)/N;

lambda_r = beta_r*(Iar + Isr)/N;

%% SYSTEM EQUATIONS

dS = Lambda - lambda_w*S - lambda_r*S + omega*R - mu*S;

dEw = lambda_w*S - (sigma_w + mu)*Ew;

dEr = lambda_r*S - (sigma_r + mu)*Er;

dIaw = p_w*sigma_w*Ew - (gamma_w + mu)*Iaw;

dIsw = (1-p_w)*sigma_w*Ew + gamma_w*Iaw - (tau_w + mu)*Isw;

dIar = p_r*sigma_r*Er - (gamma_r + mu)*Iar;

dIsr = (1-p_r)*sigma_r*Er + gamma_r*Iar - (tau_r + mu)*Isr;

dT = tau_w*Isw + tau_r*Isr - (delta + mu)*T;

dR = delta*T - (omega + mu)*R;

dXdt = [dS; dEw; dEr; dIaw; dIsw; dIar; dIsr; dT; dR];

end

**Note on MATLAB Code Used to Generate Figures**

The MATLAB codes developed for Model (1) serve as a practical computational tool for simulating the transmission dynamics of drug-sensitive and drug-resistant malaria and for reproducing the graphical results presented in Figures 4(a–f), 5(a–d), and 6(a–b). The implementation consists of a main script and a separate function file that defines the system of nonlinear ordinary differential equations. In the main script, parameter values obtained from Table 4 are assigned, and appropriate initial conditions are specified for all epidemiological compartments, including susceptible, exposed, infectious (both asymptomatic and symptomatic for wild-type and resistant strains), treated, and recovered populations. The MATLAB solver ode45, which is based on a Runge–Kutta numerical integration method, is then used to compute approximate solutions of the system over a specified time interval.

The solutions obtained are subsequently used to generate the required figures. For Figures 4(a–f), the code plots the time evolution of selected compartments to illustrate the progression of the disease and the interaction between the wild-type and resistant strains within the population. Figures 5(a–d) are produced by plotting treatment-related and recovery compartments, as well as the total population size, thereby providing insight into the effectiveness of treatment strategies and overall system behavior. To obtain Figures 6(a–b), the code performs a sensitivity analysis by varying key parameters, particularly treatment rates, and re-running the simulations to observe how these changes influence infection dynamics. By comparing multiple simulation curves on the same axes, the impact of parameter variations on both wild-type and resistant infections is clearly demonstrated.
